# Alcohol and Head and Neck Cancer: Updates on the Role of Oxidative Stress, Genetic, Epigenetics, Oral Microbiota, Antioxidants, and Alkylating Agents

**DOI:** 10.3390/antiox11010145

**Published:** 2022-01-11

**Authors:** Giampiero Ferraguti, Sergio Terracina, Carla Petrella, Antonio Greco, Antonio Minni, Marco Lucarelli, Enzo Agostinelli, Massimo Ralli, Marco de Vincentiis, Giammarco Raponi, Antonella Polimeni, Mauro Ceccanti, Brunella Caronti, Maria Grazia Di Certo, Christian Barbato, Alessandro Mattia, Luigi Tarani, Marco Fiore

**Affiliations:** 1 Department of Experimental Medicine, Sapienza University of Rome, 00185 Rome, Italy; giampiero.ferraguti@uniroma1.it (G.F.); sergio.terracina@uniroma1.it (S.T.); marco.lucarelli@uniroma1.it (M.L.); 2 Institute of Biochemistry and Cell Biology, IBBC—CNR, 000185 Rome, Italy; carla.petrella@cnr.it (C.P.); mariagrazia.dicerto@cnr.it (M.G.D.C.); christian.barbato@cnr.it (C.B.); 3 Department of Sense Organs, Sapienza University of Rome, 00185 Rome, Italy; antonio.greco@uniroma1.it (A.G.); antonio.minni@uniroma1.it (A.M.); enzo.agostinelli@uniroma1.it (E.A.); massimo.ralli@uniroma1.it (M.R.); marco.devincentiis@uniroma1.it (M.d.V.); 4 Department of Public Health and Infectious Diseases, Sapienza University of Rome, 00185 Rome, Italy; giammarco.raponi@uniroma1.it; 5 Department of Odontostomatological and Maxillofacial Sciences, Sapienza University of Rome, 00185 Rome, Italy; antonella.polimeni@uniroma1.it; 6 SITAC, Società Italiana per il Trattamento dell’Alcolismo, 00184 Rome, Italy; mauro.ceccanti@uniroma1.it; 7 SIFASD, Società Italiana Sindrome Feto-Alcolica, 00184 Rome, Italy; 8 Department of Human Neurosciences, Sapienza University of Rome, 00185 Rome, Italy; brunella.caronti@uniroma1.it; 9 Ministero dell’Interno, Dipartimento della Pubblica Sicurezza, Direzione Centrale di Sanità, Centro di Ricerche e Laboratorio di Tossicologia Forense, 00185 Rome, Italy; alessandro.mattia@poliziadistato.it; 10 Department of Pediatrics, Sapienza University Hospital of Rome, 00185 Rome, Italy; luigi.tarani@uniroma1.it

**Keywords:** oral microbiota, alcohol, alkylating agents, epigenetics, growth factors, microenvironment, oxidative stress, polyphenols

## Abstract

Head and neck cancer (HNC) concerns more than 890,000 patients worldwide annually and is associated with the advanced stage at presentation and heavy outcomes. Alcohol drinking, together with tobacco smoking, and human papillomavirus infection are the main recognized risk factors. The tumorigenesis of HNC represents an intricate sequential process that implicates a gradual acquisition of genetic and epigenetics alterations targeting crucial pathways regulating cell growth, motility, and stromal interactions. Tumor microenvironment and growth factors also play a major role in HNC. Alcohol toxicity is caused both directly by ethanol and indirectly by its metabolic products, with the involvement of the oral microbiota and oxidative stress; alcohol might enhance the exposure of epithelial cells to carcinogens, causing epigenetic modifications, DNA damage, and inaccurate DNA repair with the formation of DNA adducts. Long-term markers of alcohol consumption, especially those detected in the hair, may provide crucial information on the real alcohol drinking of HNC patients. Strategies for prevention could include food supplements as polyphenols, and alkylating drugs as therapy that play a key role in HNC management. Indeed, polyphenols throughout their antioxidant and anti-inflammatory actions may counteract or limit the toxic effect of alcohol whereas alkylating agents inhibiting cancer cells’ growth could reduce the carcinogenic damage induced by alcohol. Despite the established association between alcohol and HNC, a concerning pattern of alcohol consumption in survivors of HNC has been shown. It is of primary importance to increase the awareness of cancer risks associated with alcohol consumption, both in oncologic patients and the general population, to provide advice for reducing HNC prevalence and complications.

## 1. Introduction

Worldwide, head and neck cancer (HNC) accounts for more than 890,000 cases and 450,000 deaths annually [1]. Head and neck cancer is a malignancy, associated with the advanced stage at presentation and heavy outcomes (mean 5-year survival <50%), that occurs more often in the lips and oral cavity, nasopharynx, oropharynx, hypopharynx, and larynx; squamous cell carcinoma (SCC) represents the prevalent histology [2,3].

Alcohol abuse may result in significant mental [4,5,6,7,8] or physical health problems [9,10,11,12]. Furthermore, when consumed during gestation, it may induce severe damage to the newborns [13,14,15,16,17,18,19,20]. Alcohol is a well-known carcinogen compound but it is still underestimated in the general population, partially also because of the alcohol industry’s extensive misrepresentation of evidence about the alcohol-related risk of cancer [21,22]. Alcohol drinking, together with tobacco smoking, and human papillomavirus (HPV) infection (Table 1) are HNC-recognized risk factors [23,24,25,26]. Interestingly, the role of alcohol in HNC seems to be broader than that of a simple risk factor, as suggested from recent findings which highlighted how significant inverse association exists between alcohol drinking and prognosis among HNC patients [27,28]. It has been reported that, in 2012, a total of 203,511 cases of the oral cavity, oropharyngeal, hypopharyngeal, and larynx cancer were attributable to alcohol consumption (179,559 men and 23,952 women) [29]. The proportion of HNC cases attributable to alcohol is still increasing, emphasizing the importance of alcohol consumption limitation to prevent HNC. Alcohol use among HNC survivors negatively impacts patient outcomes and is an important risk factor for recurrent and second primary tumors. Despite recommendations from several cancer societies, alcohol consumption remains a common problem in this population. [30]. The estimate of the real alcohol consumption (based not only on what the patient declared during the anamnesis) would be of support in consolidating the correlation with the onset of HNC.

This report aims to provide a summary and subsequent review of past studies, which highlights the evidence on the role of alcohol consumption, oral microbiota, and oxidative stress in head and neck cancer onset.

## 2. Head and Neck Cancer and Alcohol

### 2.1. Diagnosis and Treatments

The HNC diagnosis usually includes laryngoscopy, imaging [Positron emission tomography/X-ray computed tomography (PET/CT) and magnetic resonance imaging (MRI)], and biopsy of the primary lesion [31,32,33,34,35]. As technology progresses, the development of non-invasive diagnostic tools in the field of head and neck oncology has been examined; the molecular analysis of tumor’s genetic features based on circulating malignance derivatives, such as circulating tumor DNA, intact circulating tumor cells (CTCs), and exosomes in patients’ blood, namely liquid biopsy, has become a concrete possible approach to improve diagnostics, treatment planning, and post-treatment surveillance in patients with the potentially curable disease [36,37,38,39].

Treatment possibilities include tumor resection (primary and/or secondary tumor), radical neck dissection, immunotherapy, radiotherapy, checkpoint inhibitors (mainly targeting the cytotoxic T-lymphocyte-associated protein 4 (CTLA-4)), programmed cell death protein 1 (PD-1), programmed death-ligand 1 (PD-L1), and chemotherapy [40,41,42,43,44,45]. Recently, it has been showing how, in locally advanced HNSCC, the CTCs and the circulating tumor microemboli (CTM) have a significant prognostic impact on the potential role as predictors of induction chemotherapy benefit [46].

It is believed that the majority of oral cancers develop from oral potentially malignant lesions (OPMLs) [47]. Though they can be easily detected during screening, risk stratification is difficult. During screening, clinicians often find it difficult to distinguish OPMLs from benign lesions, and predicting OPMLs at risk of malignant transformation could be particularly challenging [47]. DNA aneuploidy has been known to be a marker of malignancy in a number of sites, including the oral cavity [47]. Indeed, DNA ploidy and chromatin organization of cells collected from OPMLs can identify lesions at high risk of progression several years prior [48]. This non-invasive test would enable clinicians to triage high-risk OPMLs for closer follow-up, while low-risk lesions can undergo less frequent biopsies, reducing the burden on healthcare resources [48]. Quite interestingly, in a study on individuals with Fanconi anemia (people with a 500-fold to 700-fold elevated risk, much earlier onset, and limited therapeutic options for oral SCC compared with the general population), a careful inspection of the oral cavity associated with brush biopsy-based cytology could identify visible oral lesions, either malignant or potentially malignant, that warrant treatment [49].

### 2.2. Alkylating Agents

Because of the mentioned key role of genetic and epigenetic alterations in HNC, treatment protocols still include the use of alkylating agents (AAs). AAs are a heterogeneous class of drugs that interfere with the cell’s DNA and inhibit cancer cells’ growth, playing a major role in HNC [50]. These genotoxic agents modify the DNA by adding binding an alkyl group to the guanine base of DNA at the number 7 nitrogen atom of the purine ring, either directly or after metabolic conversion to reactive intermediates [51,52]. These drugs produce numerous side effects targeting many organs and apparats, such as the gastrointestinal tract, bone marrow, testicles, and ovaries; furthermore, most of the alkylating agents are also carcinogenic [53,54]. AAs still play a major role in the chemotherapeutic treatment of HNC, especially cisplatin and methotrexate, in recurrent metastatic cancer, but the focus is gradually shifting to non-conventional systemic chemotherapy, especially targeted therapy and immunotherapy, which affect the tumor microenvironment and have a potentially favorable impact on HNC management [55,56,57].

### 2.3. Alcohol Abuse Detection

**Figure 1:**
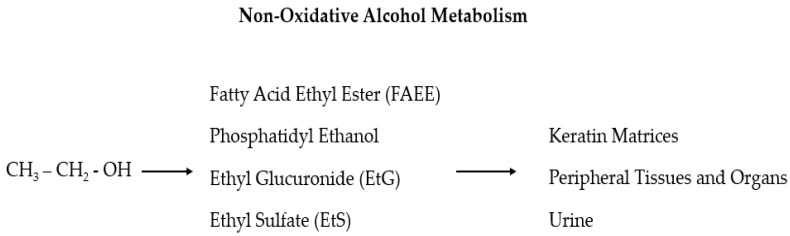
In the liver, ethanol is metabolized via oxidative and non-oxidative (less than 1%) ways. In the non-oxidative pathway, alcohol is finally processed as fatty acid ethyl ester (FAEE), phosphatidyl ethanol, ethyl glucuronide (EtG), and ethyl sulfate (EtS).

Despite the numerous proposed biomarkers in many studies, no laboratory test is sufficiently reliable alone to support a diagnosis of alcohol use disorder (AUD) [58,59]. Sensitivity and specificity should be high for alcohol abuse biomarkers, but in reality, they mostly fluctuate considerably and depend on the involved population. Furthermore, the ideal markers should reflect an individual’s consumption of alcohol, both chronically (screening markers) and acutely (relapse markers), and, from this, the given title of “state” markers (in contrast to the “trait” markers that predict the predisposition to develop alcoholism) [60,61]. The use of long-term diagnostic tools gives crucial information on the real alcohol consumption of HNC patients so that a series of recently found useful biomarkers, which can be detected in the hair, is now in the spotlight: ethyl glucuronide (EtG), fatty acid ethyl esters (products of non-oxidative ethanol metabolism), phosphatidyl ethanol, acetaldehyde adducts to protein, and 5-hydroxytryptophol [18,62,63,64]. The main advantages of this sample material are that compounds with a relatively short lifetime in blood, but with a strong correlation to alcohol consumption, can be entrapped in the hair and are detectable for a longer time (also for years depending on the length of the hair) and at a relatively high concentration [64,65]. In particular, EtG and ethyl sulfate (EtS) are two non-oxidative ethanol metabolites (Figure 1) secreted by the liver which are mainly used as markers of alcohol intake related to incidents [66,67,68,69,70,71]. These two markers for recent alcohol intake can be detected in the blood for approximately 10 h after a small to moderate alcohol intake and up to 5 days after large and repeated alcohol intakes [67,68,69]. As the efficacy of these two tests has been demonstrated in multiple settings, it has been also suggested that EtG and EtS should be included in screening tests for injured or at-risk for alcohol abuse people (including pregnant women) to investigate the possible association between residual alcohol effects and injuries, and to verify alcohol abstinence in cases of substance-related disorders [72,73].

## 3. Head and Neck Cancer Etiopathogenesis

The main risk factors for HNC are tobacco, alcohol, HPV (especially 16, for oropharyngeal carcinoma), Epstein-Barr virus (EBV, for nasopharyngeal carcinoma), and HIV/AIDS; however, an association with hepatitis C and hepatitis B infection, as well as with pro-inflammatory diet (rich in red meat and fried foods) [74,75,76,77,78], has also been observed. Interestingly, evidence suggests a link between the renin–angiotensin system and HNC, and a recent study involving 5000 patients demonstrated that angiotensin II receptor blockers usage is associated with improved overall survival (OS) and cancer-specific survival among HNC patients with chronic kidney disease or hypertension [79]. It has been demonstrated that the combination of alcohol and smoking increases the risk in a synergic way, so that the probability of HNC onset increases dramatically when these two factors coexist [80,81,82,83]. On the other hand, the beneficial impact of cessation of alcohol consumption and tobacco smoking, as well as the protective effect of fruit and salad intake, which may modulate the deleterious effects of tobacco and alcohol, has been demonstrated [84,85,86,87,88]. Recently, it has been suggested that non-smoking and non-drinking oral SCC patients may represent a different entity with a limited role for HPV infection in carcinogenesis, but also that they are associated with worse outcomes when expressing HPV16 [89,90].

### 3.1. Genomic Alterations

The tumorigenesis of HNC represents an intricate sequential process that, while progressing from squamous hyperplasia through graded dysplasia to invasive carcinoma, implicates a gradual acquisition of genetic and epigenetic alterations, with genetic damage and repair and chromosomal loss and gain targeting the critical components of crucial genetic pathways regulating cell growth, motility, and stromal interactions [91,92]. The accumulation of alterations in crucial tumor suppressor genes (such as TP53 and CDKN2A) or signaling pathways (such as PI3K–AKT–mTOR and RAS–MAPK) is associated with the onset, progression, and poor prognosis of HPV-negative HNCs [93]. The most commonly disrupted pathways in HNCs patients are those involved in tumor protein 53 (TP53) and retinoblastoma (RB) activity, with wild-type mutations more commonly seen in HPV-negative patients [94,95,96]. The TP53 gene is a tumor suppressor transcription factor that regulates the cell cycle, preventing cell growth and promoting cell apoptosis in the presence of DNA damage. Over 50% of the HNCs patients with TP53 pathway alterations present chromosomal loss at 17p (the site where the TP53 gene resides) [97,98].

The RB pathway is mostly disrupted by the inactivation resulting mainly from promoter hypermethylation, gene mutation, or loss of heterozygosity (LOH) of p16^INK4A^, a tumor suppressor protein that inhibits cyclin-dependent kinase (CDK) 4/6 and prohibits cells from entering the cell cycle. Up to 80% of HNSCCs present LOH at chromosomal region 9p21, where p16^INK4A^ resides [99,100,101,102]. The HPV-positive patients do not require the inactivation of p16^INK4A^ to have RB inhibition because the expression of the viral protein E7 causes the degradation of RB protein [103]. The HPV-positive tumors are distinctively characterized by frequent loss of TNF receptor-associated factor 3 (TRAF3) and amplification of E2F transcription factor 1 (E2F1), while HPV-negative tumors often present CDKN2A and TP53 alterations; focal deletions in other tumor suppressor genes (such as NSD1, FAT1, NOTCH1, and SMAD4); and frequent focal amplification of the genes encoding the EGFR, HER2, and FGFR1 receptor tyrosine kinases [104].

### 3.2. Tumor Microenvironment

Recently, the importance of the tumor microenvironment (TME) [105,106,107,108] has been emphasized, namely the result of factors associated with cancer, patient’s characteristics (such as oral cavity microbiota, see paragraph below), immune system, and factors related to geographic origin, specifically embodied by the complex and dynamic interactions among the various cells as well as the balance of proangiogenic factors, tissue pH, growth factors, and cytokine production changes over time [109,110]. Typically, HNC-TME is characterized by hypoxia (related to poor prognosis and resistance to radiation therapy) and immune cells infiltration, while an active tumor–stromal cross-talk is essential to promote cancer growth and invasion, with an important role played by cancer-associated fibroblasts (CAFs), chemokines, cytokines, and proliferative and inflammatory signal pathways [111,112,113]. The production of growth factors, such as vascular endothelial growth factor (VEGF), by both tumor cells and CAFs, causes the recruitment of endothelial cells which stimulate the neovascularization needed to bring oxygen and nutrients to sustain the tumor supporting the survival and self-renewal of cancer stem cells (CSCs) [93,114]. Meanwhile, IL-10 and IL-1 induce interferon-γ (IFN-γ) downregulation, which stimulates matrix metalloproteinases (MMPs) to support metastatic tumor cells’ escape and angiogenesis [115].

### 3.3. Growth Factors

Many growth factors and their receptors have been extensively studied for both their role in HNC pathophysiology and possible target therapy development. One of the most studied is the epidermal growth factor receptor (EGFR) [116,117,118,119,120], a transmembrane tyrosine kinase receptor of the ErbB family that promotes multiple signaling pathways involved in tumor cell growth, evasion, angiogenesis, and invasion [121]. The EGFR activation in HNC is mainly driven by the high expression of its soluble ligands (EGF and transforming growth factor-alpha (TGF-α)), resulting in the dimerization of EGFR, the autophosphorylation of its intracellular kinase domain, and the stimulation of a proliferative and pro-survival intracellular signaling through the mitogen-activated protein kinase (MAPKs) cascade (as well as the PI3K-AKT-mTOR and JAK-STAT pathways) [122]. Even though more than 90% of HNCs overexpress EGFR (the expression progressively increases according to the histological malignant transformation, from hyperplasia to invasive carcinoma), which is associated with high local recurrence rate and poor survival, only a modest subgroup of HNCs shows amplified copy numbers or mutational activation of the EGFR gene, suggesting the existence of other mechanisms acting downstream on the pathways [123]. Targeted therapy specifically directed towards EGFR has been an area of keen interest in head and neck cancer research, as EGFR is potentially an integration point for convergent signaling [124]. However, despite the latest advancements in cancer diagnostics and therapeutics against EGFR, the survival rates of patients with advanced head and neck cancer remain disappointing due to the resistance to anti-EGFR therapies [124]. Interestingly, it has been demonstrated that G protein-coupled receptors (often overexpressed in HNC) ligands prostaglandin E2, bradykinin, and gastrin-releasing peptide activate EGFR signaling [125,126,127]. Another interesting therapeutic and prognostic target is the VEGF pathway (a member of the platelet-derived growth factor, PDGF, superfamily) [128,129,130,131,132,133,134,135], whose receptors are expressed both on endothelial cells and tumor cells and that include VEGF from A to E. VEGF (mainly VEGF-A) is often overexpressed in HNC and plays a key role as a mediator of angiogenesis, which supports tumorigenesis potentially modulating the tumor microenvironment [136,137]. On the other hand, hypoxia, one of the main components of the HNC microenvironment, is a major factor inducing VEGF expression through the expression of hypoxia-inducible factor-α (HIF-1α) [138,139]. The main angiogenesis downstream signal in HNC is usually mediated by VEGF-A and VEGFR-2; VEGF signaling can promote tumor cell proliferation, migration, immune system evasion, and cancer invasion (by activating key pathways such as the MAPK and PI3K–AKT), and can also play a role in chemotherapy resistance as it may induce autophagy that counteracts chemotherapy-induced stress [140,141]. Fibroblast growth factor receptor (FGFR) is often overexpressed in HNC, especially FGFR1, which is more commonly altered in the larynx and hypopharynx primary tumor locations. This plays an important role in the proliferation, survival, and migration of cancer cells, as well as angiogenesis [142,143,144,145]. Interestingly, it seems that ethanol-induced growth factors alterations are partially related to the oxidant capacity of the beverage, so that when ethanol is administered alone, it has different effects than when consumed in drinks with antioxidant properties (e.g., red wine) [146,147].

Neurotrophins and their receptors have a nerve growth factor (NGF) which plays key roles in immune disorders [148,149,150], nerve cells growth and development [151,152,153], cardiovascular disorders [154,155,156], and psychiatric diseases [157,158,159,160]. Furthermore, it might regulate cell survival in HNC patients.

In particular, it has been demonstrated that the p75 neurotrophin receptor, the low-affinity receptor of nerve growth factor (NGF), is abnormally expressed in HNC patients and is related to NGF-independent therapy resistance, while high-affinity NGF receptor (tropomyosin receptor kinase A, TrkA) might transduce a survival signal of NGF, stimulating tumor cell survival after cell cycle arrest [161,162,163,164]. Interestingly, it has been demonstrated that alcohol drinking can modulate neurotrophins expression, causing both genetic and epigenetic effects [164,165].

## 4. Cancerogenic Effects of Alcohol

**Figure 2:**
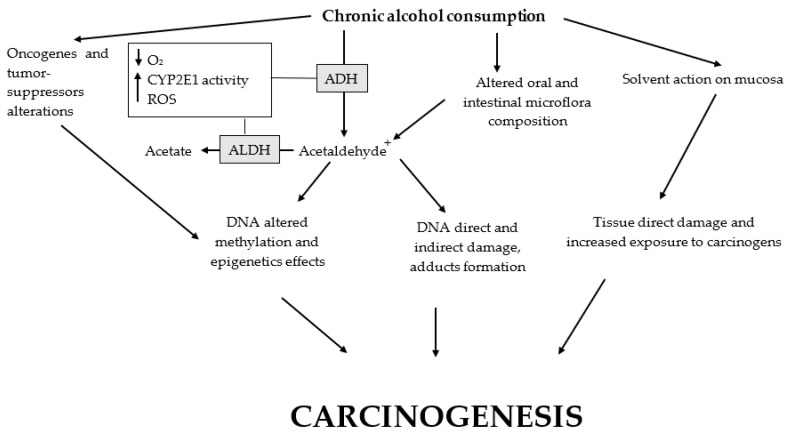
Main alcohol carcinogenic effects. Altered oral cavity and large intestine microflora, as well as polymorphisms of CYP2E1, ADH, and ALDH, promote the carcinogenic effects of alcohol. Ethanol chronic ingestion causes: (1) microflora changes with a reduction in the protective bacteria and prevalence of the flora related to increased production of acetaldehyde and its toxic effects; (2) the development of a hypoxic microenvironment with increased levels of ROS partially due to the activity of CYP2E1 (consequentially, ROS cause direct and indirect damage to macromolecules and DNA); (3) both increased oxidative stress and acetaldehyde metabolism may cause the formation of DNA adducts; (4) alcohol metabolism to acetaldehyde is related to altered DNA methylation with epigenetic effects; (5) somatic copy-number alterations of oncogenes and tumor suppressors may be triggered; and (6) a direct solvent action on the oral mucosae causing tissue damage and exposing the epithelial cells to carcinogens. ADH, alcohol dehydrogenase; ALDH, aldehyde dehydrogenase; CYP2E1, cytochrome P450 2E1; ROS, reactive oxygen species.

Alcohol is a known carcinogen and an independent risk factor for HNC but, when evaluating the relationship between alcohol and HNC (Figure 2), it is particularly important to take more account of those relevant studies that consider the risks from alcohol consumption in lifelong non-smokers to reduce the issues related to the effects of tobacco smoking [82,166]. Both smoke and alcohol exposure causes DNA damage and inaccurate DNA repair [93]. An etiological role for both ethanol and its primary oxidative metabolite acetaldehyde has been shown; in particular, the most relevant cancers considered to be causally related to alcoholic beverage consumption include those of the upper aerodigestive tract (oral cancer and cancers of the oropharynx, hypopharynx, larynx, and esophagus), the liver, the colon, the rectum, and the breast, with the greatest evidence for oral cavity, pharynx, esophagus, and larynx cancers [167,168]. While some papers reported that acetaldehyde rather than ethanol is the most important carcinogenic, i.e., a tumor initiator by binding of DNA and formation of DNA adducts, it has actually been proven that alcohol toxicity is caused both directly by ethanol and indirectly by its metabolic products, including the reactive oxygen species (ROS) produced during its biotransformation involving CYP2E1 [169,170,171]. It has been proposed that, regarding HNCs, alcohol might enhance the exposure of epithelial cells to carcinogens, but a variety of pathophysiological biomechanisms have also been linked to the direct or indirect tumorigenic effects of alcohol (oxidative stress, epigenetic modifications, DNA damage, inaccurate DNA repair, and the formation of DNA adducts) [121,172]. Furthermore, alcoholic beverages contain many different substances derived from fermentation (such as ethyl carbamate) which have proven to be carcinogenic to humans [59,173]. It has been demonstrated that heavy alcohol consumption may trigger somatic copy-number alterations of oncogenes and tumor suppressors frequently associated with HNC mutations (CDKN2A, FHIT, 11q13 region, HER2, 3q25-qter, and CSMD1) but, inversely to tobacco, it may not induce TP53 mutation [174]. Aldehyde dehydrogenase 1 (ALDH1), a component of alcohol metabolism major pathway located at chromosome 9q21.13, is considered a highly selective prognostic marker in HNC; indeed, ALDH1+ cells displayed resistance to radiotherapy and could generate tumors [175]. Unfortunately, there is no clear threshold effect of alcohol for both neoplasms and several non-neoplastic diseases [176].

### 4.1. Oxidative Stress

Physiologically, alcohol is mainly oxidized by the enzymes alcohol dehydrogenase (ADH), cytochrome P-450 2E1 (CYP2E1), and catalase to form acetaldehyde, which is subsequently oxidized by ALDH2 to produce acetate [177]. Among the ADH family, ADH1B plays a major role in ethanol oxidation, and polymorphisms in the ADH1B gene have been associated with upper aerodigestive tract cancer in Caucasians and the Japanese population, while individuals presenting with the ADH1B*2 allele (commonly observed in Asian population) show much higher (about 40 times) enzymatic activity than those with the wild-type ADH1B*1 allele, resulting in high acetaldehyde exposure but lower risk of cancer because of the “flushing response” to alcohol associated with facial flushing, palpitations, and headaches, as well as other unpleasant symptoms that prevent them from heavy drinking [178,179,180,181]. Similarly, and often concurrently, the enzyme encoded by ALDH2*2 (also common in the Asian population) has a lysine for glutamate substitution at residue 487, resulting in a loss of activity so that ALDH2*2/*2 homozygotes (and to a lesser extent in ALDH2*1/*2 heterozygosis) exhibit a flushing response to alcohol that prevent them from alcohol drinking [182,183,184]. The induction of CYP2E1 expression is one of the key pathways whereby ethanol can lead to the generation of ROS, altering the intracellular redox state, and ultimately leading to a global increase in oxidative stress and neuronal cells death by the oxidation of proteins, lipids, and DNA [185,186]. ROS (superoxide, hydrogen peroxide, hydroxyl radicals, and singlet oxygen) can oxidize cellular DNA, leading to several alterations, including oxidized bases, single-/double-stranded breaks, and the generation of oxidative DNA adducts, which can cause genetic mutations resulting in cellular immortalization and clonal expansion, ultimately leading to cancer [104]. Interestingly, it has been found that the frequently found mutations in the genes encoding nuclear factor erythroid 2-related factor 2 (NFE2L2) and Kelch-like ECH-associated protein 1 (KEAP1), i.e., key regulators of oxidative stress, occur exclusively in HPV-negative HNC [104]. As mentioned, ROS attack all cellular macromolecules, not only DNA, and in particular, they initiate lipid peroxidation and damage to cell membranes producing crotonaldehyde, acrolein, 4-hydroxy-2-nonenal, and malondialdehyde, which are reactive substances that damage DNA through the formation of exocyclic adducts [187,188,189]. Another possible cause of oxidative stress is the mitochondrial dysfunction associated with alcohol chronic ingestion [190,191]. Increased activity of the enzyme nicotinamide adenine dinucleotide phosphate oxidase (NOX) is a key source of ROS production so that co-treatment with NOX inhibitors is considered useful to prevent ethanol-induced increases in NOX activity, ROS generation, and oxidative DNA damage [190,192,193]. In murine experiments, it has been demonstrated that antioxidant vitamin E (alpha-tocopherol) adjusts the levels of anti-apoptotic and pro-apoptotic proteins and corrects organs alterations and DNA damage [194,195,196,197].

Many other antioxidants have shown promising therapeutic effects against alcohol pro-oxidative and mutagenic action, mainly in rodents, including ascorbic acid (vitamin C), beta-carotene, black ginseng, EUK-134 (synthetic superoxide dismutase plus catalase mimetic), folic acid, melatonin, N-acetylcysteine, phenyl butyl nitrone, pycnogenol, silymarin, resveratrol, hydroxytyrosol, and superoxide dismutase [185,198,199,200,201,202,203,204,205,206,207].

### 4.2. Oral Microbiota

Recent scientific investigations have focused on studying the role of the oral microbiota in the pathogenesis of upper respiratory tract tumors [208,209,210,211,212]. While it is clear that the presence of localized infections can negatively impact the outcome of oral cancer, it is not equally clear whether or not the existence of dysbiosis is, in itself, a cause or consequence of the presence of the tumor. This is mainly due to the fact that a few studies analyze the modification of the microbial population in the early stages of tumor onset [213,214]. As regards the impact of alcohol consumption on the microbial population of the oral cavity, a large study conducted on the American population has shown that, especially in heavy drinkers, there is an alteration of the microbial composition [215]. In particular, there is a significant decrease in lactobacilli, whose presence is associated with beneficial, anti-inflammatory, and antioxidant effects. In particular, in the oral cavity, the administration of probiotics based on *Lactobacilli* has been shown to inhibit the proliferation of pathogens and reduce gingival inflammation [216,217].

As for the interaction between alcohol, oral microbiota, and laryngeal tumors, certain studies highlight the potential role of some microorganisms in mediating the carcinogenic effect of alcohol [218,219]. Indeed, acetaldehyde, the main product of alcohol metabolism, with proven carcinogenic effects, seems to be produced locally by the action of microorganisms, including bacteria and yeasts. Acetaldehyde formation has been described in human mouth washings and bronchopulmonary washings in vitro, and it was reduced by using antibiotics, suggesting an oral bacterial origin [220,221]. Among the bacterial species normally habiting the oral cavity, genus *Neisseria* has particularly high ADH activity and produces significant amounts of acetaldehyde when cultured in the presence of ethanol in vitro [222]. The ability to produce acetaldehyde was more than 100-fold higher than that produced by any other genera studied. Additionally, alcohol ingestion influences the microbial composition of the oral cavity, resulting in an augmented amount of *Neisseria*. Although *Neisseria* present in the ordinary oral microflora is non-pathogenic, these results suggest that it could be a local source of carcinogenic acetaldehyde and, thus, potentially participates in the alcohol-related carcinogenesis in human oral cancer. Among *Neisseria* species, *N. subflava* isolated from patients has shown ADH activity and the ability to produce acetaldehyde from ethanol [222]. A recent study conducted in Japan explored the salivary microbiota profiles of healthy adults in order to evaluate the acetaldehyde production in the oral cavity [223]. A marked difference in the salivary acetaldehyde production ability, depending on the oral microbiota of healthy adults was found, but this was independent of the abundance of *Neisseria* species in the salivary microbiota. These data confirm the ability of *Neisseria* spp. to locally metabolize ethanol [223].

Acetaldehyde production from ethanol by oral streptococci has been also demonstrated in the study conducted by Kurkivuori et al. [222]. Authors demonstrated that diverse strains showed a significantly different ability to metabolize ethanol, as demonstrated by experiments conducted by using *Streptococcus salivarius*, *Streptococcus intermedius,* and *Streptococcus mitis* that produced high amounts of acetaldehyde, from different ethanol concentrations. Based on such pieces of evidence, these bacteria could be considered dangerous in heavy drinkers [224,225].

Finally, the production of carcinogenic acetaldehyde by *Candida* spp. has been suggested. In particular, both *Candida albicans* and *non-albicans* contribute to epithelial dysplasia and oral carcinogenesis, by producing mutagenic amounts of acetaldehyde, from glucose and ethanol [226,227,228,229]. Indeed, an interesting paper by Nieminen and co-workers demonstrated the ability of *Candida glabrata* to produce acetaldehyde, from glucose and ethanol, supporting a role in the development of oral cancer [230].

### 4.3. Acetaldehyde DNA Adducts

Alcohol and tobacco carcinogens and their metabolites can bind covalently to DNA, resulting in the formation of DNA adducts [231,232,233,234,235,236,237] which, if unrepaired, can cause miscoding and permanent mutations. These can then activate oncogenes or inactivate tumor suppressor genes driving the cancerogenesis process (it may be a major initiating event of chemical carcinogenesis) [238,239,240]. The main DNA adduct in the human body is a Schiff base N2-ethylidene-2-deoxyguanosine adduct (which may be converted by reducing agents to N2-ethyl-2’-deoxyguanosine altering DNA synthesis), though products are also observed in reactions with deoxyadenosine (dA) and deoxycytidine (dC) [241,242]. As mentioned, polymorphisms of the genes which encode enzymes (e.g., ALDH2) for alcohol metabolism impact ethanol oxidizing capacity, leading to the accumulation of acetaldehyde as well as an increased risk for developing alcohol-induced complications, such as HNC [243]. The key mechanism for alcohol-induced adducts involves acetaldehyde, whose cancerogenic effects are connected to multiple paths; it interferes with DNA synthesis and repair, causes mucosa lesions, inhibits O6-methyl-guanylyltransferase (an enzyme important for the repair of adducts caused by alkylating agents), and covalently binds to DNA-forming adducts [244,245]. Despite the interest, it is still unclear whether these alcohol-related DNA adducts are true factors or initiators of cancer, but it has been suggested that measuring aldehyde-induced DNA and protein adducts produced during alcohol metabolism may allow earlier detection of alcohol abuse disorders and complications [246].

### 4.4. Epigenetics

Epigenetics refers to the “study of heritable changes in gene expression that occur without a change in the DNA sequence” so that epigenetic mechanisms consent to an ulterior transcriptional control that regulates how genes are expressed [247,248,249,250]. In addition to genetic alterations, epigenetic changes (mainly DNA methylation, histone methylation, and deacetylation) also play an important role in driving HNC oncogenesis [251,252,253,254,255,256,257]. On the other hand, epigenetic mechanisms are strongly related to alcoholism and alcohol drinking disorders [165,258,259]. In HNC, DNA hypermethylation causes epigenetic silencing of tumor suppressor genes (especially those involved in DNA repair, cell cycle control, apoptosis, angiogenesis, cell–cell interaction, and metastasis) which is considered a specific marker of cancer [250,260,261]. On the other hand, despite the presence of regional promoter hypermethylation, HNC reveals global genomic hypomethylation in about 67% of cases, and the degree of global hypomethylation (measured by the level of methylation of repetitive sequences across the genome) is associated with smoking history, alcohol use and tumor stage [262,263]. It has been demonstrated that the etiologic heterogeneity of HNC is reflected in specific patterns of molecular epigenetic alterations within the tumors and that the DNA methylation profiles and gene expression may hold clinical potential [264,265]. Indeed, gene expression profiles are strongly associated with the development of HNC, and DNA methyltransferase 3B (DNMT3B, a de novo DNA methyltransferase) polymorphism has been identified as one of the most important factors associated with these tumors because of its connection with CpG island methylator phenotype (CIMP), a possible early event during HNC development [251,266,267]. Studies evaluating increased promoter methylation, as well as the resulting downregulated expression of key tumor suppressor genes from normal mucosa to premalignant lesions and HNC, included many genes such as cyclin-dependent kinase inhibitor 2A (CDKN2A), retinoic acid receptor beta (RARβ, which mediates epithelial cell differentiation), deleted in colorectal cancer netrin 1 receptor (DCC), O-6-Methylguanine-DNA methyltransferase (MGMT, involved in the repair of DNA damage from tobacco carcinogens), p16^Ink4^, p14^ARF^, endothelin receptor type B (EDNRB), E-cadherin (CDH1), deleted in lung and esophageal cancer 1 (DLCE1), and NDRG family member 2 (NDRG2) [255,268,269,270,271]. Interestingly, it has been demonstrated that IL-6-induced inflammation promotes HNC tumorigenesis by altering global long interspersed nuclear element-1 (LINE-1) hypomethylation, while concurrent hypermethylation of multiple tumor suppressor genes by IL-6 suggests that epigenetic gene silencing may be a vital consequence of head and neck tissues chronic inflammation [272].

## 5. Strategies for Prevention

### Polyphenols

It has been demonstrated that the content of antioxidants (polyphenols) in alcoholic drinks (i.e., resveratrol in the red wine) may counteract the toxic effect of alcohol in animal models [198,199,273,274,275,276,277,278,279]. In particular, polyphenols are natural, synthetic, or semi-synthetic organic molecules characterized by several hydroxyl groups on aromatic rings (phenolic groups), presenting neuroprotective effects and a capacity to control oxidative stress, inflammation, apoptosis, and mitochondrial dysfunction. They can be divided into four main groups: phenolic acids, flavonoids, stilbenes, and lignans [274,280,281]. The Mediterranean diet is a mainstay of nutritional therapeutic and preventive programs in HNC because of the rich presence of foods abundant in polyphenols, such as olives and olive oil, as well as fresh and processed fruits and vegetables, leguminous plants, cereals, herbs, spices, tea, coffee, wine, and beer [282,283,284,285]. A proper diet is one of the major factors contributing to good health and is directly related to the general condition of the organism [273,286]. Polyphenols are converted and absorbed mainly in the oral cavity and stomach; in the large intestine, the remaining polyphenols are further modified by bacterial enzymes (e.g., glycosides, esters, etc.) to obtain lower-weight metabolites which are easier to absorb. The metabolites then circulate within the blood, bind to proteins (mainly albumin), and conjugate in the liver and kidneys, before finally being eliminated in urines and feces [287]. Plant polyphenols are natural antioxidants which, at the same time, also exhibit prooxidant properties (also important for polyphenols anticancer properties), catalyzing oxidative DNA cleavage particularly in the presence of transition metal ions such as copper and iron [199,288]. Polyphenols in oils, especially extra virgin olive oil, are effective in both preventing cancer and in reducing toxicity and carcinogenicity of alcohol, mainly due to its antioxidant properties [276,289,290,291]. In an dimethyl-benzanthracene-induced hamster model of carcinogenesis, green tea lowered detectable tumors by almost 35% and tumor volume by almost 57%, while tea (*Camellia sinensis*) constituents inhibited carcinogenesis of various tumors, including HNC, by inducing apoptosis and reducing cell proliferation, with a major role played by epigallocathechin-3 gallate (ECG) and theaflavins through inhibiting mitogen-activated protein kinases, as well as signaling growth factor, cyclin-dependent kinases, including topoisomerase-1 and many other potential targets [292,293].

Polyphenols are also present in alcoholics, such as red wine (whose main polyphenol is resveratrol) and beer, with evidence that moderate wine consumption may decrease the risk of several cancers, including colon, basal cell carcinoma, ovarian (while breast cancer is promoted), and prostate cancer; however, it is essential to maintain adequate balance to avoid the negative and cancerogenic effects due to the ethanol present in these beverages, mainly because these results are obtained from studies on human cancer cells in culture, murine models, or as conclusions from epidemiological studies, rather than from clinical trials with cancer patients [294,295]. The wine may inhibit carcinogenesis by acting as an antioxidant, anti-inflammatory, antimutagen, antimetastatic, anti-angiogenic, antidifferentiation, antiproliferative, and proapoptotic agent which can modulate signal transduction, immune response, transcription factors, growth factors, cytokines, caspases, interleukins (ILs), prostaglandin synthesis, and cell cycle-regulating proteins [296].

## 6. Discussion

In this narrative review, we analyzed literature evidence concerning the role of alcohol consumption in HNC onset. Though alcohol is not the sole risk factor for HNC, it plays a major role in the etiopathogenesis of both primary tumors and their recurrences. Alcohol carcinogenicity is mainly caused both directly by ethanol and indirectly by its metabolic products; it enhances intracellular oxidative stress and the exposure of epithelial cells to carcinogens, and is associated with epigenetic mutations, DNA damage, and inaccurate DNA repair related to the formation of DNA adducts [169,170,171,172,258]. Alcohol consumption may trigger somatic copy number alterations of oncogenes and tumor suppressors which are frequently associated with HNC mutations [174]. Unfortunately, there is no clear threshold effect of alcohol for oncogenic patients [176].

Since the relationship between alcohol and HNC has been largely established, long-term markers of alcohol consumption, especially those detected in the hair, can give crucial information on the real alcohol drinking habits of HNC patients [63,64]. Furthermore, many prognostic markers related to alcoholism, especially those linked to the polymorphisms of ethanol metabolic pathway components, have been suggested for the detection and monitoring of HNC [109]. With this knowledge on the etiopathogenesis of HNC and its relation to alcohol-induced oxidative stress and genetic-epigenetic alterations, more attention could be focused on the role of polyphenols and alkylating agents for patient management, especially in the case of heavy drinkers [188,279,287].

## 7. Conclusions

Alcohol abuse is a dangerous condition which affects both females and males, causing significant dangerous effects, especially in the long term. Despite the established association between alcohol and HNC, a concerning pattern of alcohol consumption, both in the general population and in survivors of HNC, has been shown. It is of primary importance to address the problem of alcohol drinking, both in oncologic patients and the general population, to rectify misconducts and to reduce HNC prevalence and complications.

## Figures and Tables

**Table 1:** Major differences between HPV + and HPV - HNSCC (mainly related to alcohol abuse and smoke). Alcohol is a major determinant of aggressive HNCs. HNSCC, head and neck squamous cell carcinomas; HPV, human papillomavirus.

	HPV + HNSCC	HPV - HNSCC
**Main risk factors**	Sexual contact, HPV type 16 and 18	Alcohol and smoking
**Tumor site**	Oropharynx	Non-oropharyngeal sites
**Histopathology**	Basaloid, non-keratinizing, poorly differentiated	Keratinizing, moderately differentiated
**Main carcinogenic factor**	Viral protein E6 and E7 action	DNA damage and inaccurate DNA repair promoted by alcohol catabolism and smoke carcinogen components action
**Responsiveness to chemoradiation**	Better than HPV - HNSCC	Worse than HPV + HNSCC
**Prognosis**	Better than HPV - HNSCC	Worse than HPV + HNSCC
**Prevention**	HPV vaccine, condom	Alcohol and smoking abstinence
